# Influence of Concentration Levels of β-Tricalcium Phosphate on the Physical Properties of a Dental Adhesive

**DOI:** 10.3390/nano12050853

**Published:** 2022-03-03

**Authors:** Amal S. Al-Qahtani, Huda I. Tulbah, Mashael Binhasan, Sara Shabib, Khulud A. Al-Aali, Mai M. Alhamdan, Tariq Abduljabbar

**Affiliations:** 1 Department of Prosthetic Dental Sciences, College of Dentistry, King Saud University, P.O. Box 21069, Riyadh 11475, Saudi Arabia; aalkahtany@ksu.edu.sa (A.S.A.-Q.); htulba@ksu.edu.sa (H.I.T.); mayalhamdan@ksu.edu.sa (M.M.A.); 2 Division of Operative Dentistry, Department of Restorative Dentistry, College of Dentistry, King Saud University, Riyadh 60169, Saudi Arabia; mbinhasan@ksu.edu.sa (M.B.); sashabib@ksu.edu.sa (S.S.); 3 Department of Clinical Dental Sciences, College of Dentistry, Princess Nourah Bint Abdulrahman University, Riyadh 11671, Saudi Arabia; kaalaali@pnu.edu.sa

**Keywords:** adhesive, bonding, dentin, calcium, phosphate, β-TCP

## Abstract

Our study assessed the influence of integrating 5% and 10% tricalcium phosphate (β-TCP-Ca_3_(PO_4_)_2_.) nanoparticles into a dental adhesive on the adhesive’s bonding. To evaluate the filler nanoparticles, scanning electron microscopy (SEM), Energy Dispersive X-Ray (EDX) spectroscopy, Fourier-transform infrared (FTIR) spectroscopy, and micro-Raman spectroscopy techniques were used. Shear Bond strength (SBS) testing, degree of conversion (DC) analysis, investigation of the adhesive–dentin interface, and biofilm experiments were conducted. The SEM micrographs revealed non-uniform agglomerates, while the EDX demonstrated the existence of oxygen ‘O’ (24.2%), phosphorus ‘P’ (17.4%) and calcium ‘Ca’ (60.1%) in the β-TCP nanoparticles. The FTIR and micro-Raman spectra indicated characteristic bands for β-TCP containing materials. The 10 wt.% β-TCP adhesive presented the highest SBS values (NTC-10 wt.% β-TCP: 33.55 ± 3.73 MPa, TC-10 wt.% β-TCP: 30.50 ± 3.25 MPa), followed by the 5 wt.% β-TCP adhesive (NTC-5 wt.% β-TCP: 32.37 ± 3.10 MPa, TC-5 wt.% β-TCP: 27.75 ± 3.15 MPa). Most of the detected failures after bond strength testing were adhesive in nature. The β-TCP adhesives demonstrated suitable dentin interaction by forming a hybrid layer (with few or no gaps) and resin tags. The β-TCP adhesives (10 wt.%) revealed lower DC values compared to control. The incorporation of 5 and 10 wt.% concentrations of β-TCP particles resulted in an increase in SBS values. A linear decline in DC values was witnessed when the nanoparticle concentration was increased. Further research focusing on exploring the influence of higher filler concentrations on adhesive’s properties is recommended.

## 1. Introduction

Dentin is an intrinsically humid tissue that makes adhesive bonding one of the most puzzling tasks in dentistry [1]. Adhesive dentistry has seen a paradigm shift in the last two decades, with new materials being available that are user-friendly and establish stronger adhesion with dentin as compared to conventional adhesives [2]. Still, the longevity of the adhesive–dentin bond is questionable, and it is dependent upon several factors. One essential factor is the capability of the adhesive monomers to penetrate the spaces between the collagen fibers in order to form strong resin tags, which consequently improves the stability of the adhesive–dentin bond [3]. Another key factor that impacts the durability of the adhesive–dentin bond is the composition of the adhesive that influences its various properties [4]. Hence, it has been suggested in the literature that the inclusion of inorganic nanofillers in the adhesive composition improves its bond strength, inhibiting its continuing degradation over time [5,6]. A durable adhesive–dentin bond verifies that the restoration will endure the dynamic oral environment, which regularly experiences temperature and pH fluctuations [7]. The literature shows that the addition of inorganic nanofillers in the adhesive improves its various mechanical properties [5], subsequently enhancing the longevity of the resin composite restorations [8]. The size of these nanofillers typically ranges between 1 and 100 nm, and they possess encouraging antibacterial properties (owing to their great chemical reactivity) [9,10]. As these fillers are nano-sized, they possess a larger surface area to the mass ratio, which helps them to work with negatively charged bacterial surfaces, improving the antibacterial potential of the material [11,12,13]. Although nanoparticles based on bioactive glasses, hydroxyapatite, silica, and graphene oxide have been added to adhesives previously [5], the quest to find a material that enhances various mechanical and antimicrobial properties of dentin adhesives optimally continues. One such filler material that can be used to enhance the properties of dentin adhesives is tricalcium phosphate (TCP).

Conventionally, TCP has been used for osteogenic purposes in the case of bone resorption [14]. Although this material has several polymorph phases [15], the α and β polymorphs are the most commonly used for clinical applications, including enamel remineralization, prevention of enamel demineralization, and to augment properties of dentin adhesives [16,17,18]. In comparison to the α-TCP, the β-TCP (Ca_3_(PO_4_)_2_ is comparatively less soluble and can be produced at comparatively lower temperatures, hence being cost-effective [19]. Furthermore, TCP nanoparticles are biocompatible, making them useful for several dental applications [20]. In a former study, the integration of TCP filler in the adhesive’s composition resulted in a higher polymerization rate and hardness [18]. In another earlier study, the inclusion of β-TCP nanoparticles in the adhesive’s composition caused an improvement in its bond strength without affecting the degree of conversion (DC) significantly [21]. β-TCP nanoparticles have recently gained popularity in dentistry, and it would be interesting to see the impact of their inclusion on numerous properties of dentin adhesives. Keeping in mind all these benefits of β-TCP, it is added to dental adhesive as this could possibly enhance several properties of the adhesive.

Therefore, our study was aimed at integrating 5% and 10% β-TCP nanoparticles into CA and then observing the effect of its addition on adhesive bonding and degree of conversion. It was assumed that the addition of β-TCP nanoparticles to the adhesive’s composition would improve its mechanical properties. 

## 2. Materials and Methods

Our study proposal was accepted by the ethics review board of the specialist dental practice and research institute before commencement. Non-carious and defect-free extracted maxillary third molar teeth were obtained from Oral Surgery clinics. These teeth were stored in 10% formalin and used within a month of their collection for the experiments in our study. 

### 2.1. Amalgamation of Nanoparticles with the Dental Adhesive

β-TCP nanoparticles were obtained (β-tricalcium phosphate- Sigma Aldrich, St. Louis, MO, USA) and added in two concentrations (5% and 10%, m/m) to the control adhesive (CA) (Prime and bond- Dentsply, Caulk, Tulsa, OK, USA) (di- and tri-methacrylate resins, PENTA (dipentaerythritol penta acrylate monophosphate), and acetone), to yield 5 wt.% β-TCP and 10 wt.% β-TCP adhesive groups. To guarantee homogenized dispersion of the nanoparticles in the adhesives, they were shifted in a centrifuge and then sonicated. The adhesives, which included CA (without nanoparticles), 5 wt.% β-TCP, and 10 wt.% β-TCP, were stored at 4 °C and used within three weeks of their amalgamation. 

### 2.2. Characterization of the Filler Nanoparticles

Multiple methods were used to characterize the filler nanoparticles. The morphology of the β-TCP nanoparticles was assessed using scanning electron microscopy (SEM). A small quantity of the filler nanoparticles was placed on the aluminum stubs and then covered with a gold layer. Using an SEM (JEOL JEM-1400 series 120kV- USA), these β-TCP nanoparticles were then observed. The accelerating voltage of SEM was set at 30 kV while different magnifications were used to notice the morphological features of the filler nanoparticles. Using the energy-dispersive X-ray (EDX) spectroscopy technique, elemental analysis of the newly produced β-TCP nanoparticles was carried out. We also used the Fourier-transform infrared (FTIR) spectroscopy technique to further characterize the filler nanoparticles. The filler nanoparticles were applied to the potassium bromide disc of the spectroscope. While the connection with FTIR (Thermo Scientific Nicolet IS10 FTIR spectrometer, Waltham, MA, USA) sensors was maintained, the data were collected over a range of 500–1500 cm^−1^ (wavenumber). The last characterization of the filler nanoparticles involved using micro-Raman spectroscopy. A Raman spectrophotometer (Thermo Scientific Nicolet IS10 FTIR spectrometer, Waltham, MA, USA) and Raman reader^®^ software was used to collect the spectra. The laser beam was held using an objective lens, and a power of 600 mW was used. The 1 min scans were completed three times, and the results were averaged. 

### 2.3. Tooth Specimens Preparation and Bonding Protocol 

Ninety teeth (*n* = 90) were secured in orthodontic resin (Opti-Cryl, South Carolina, Columbia) at the level of the cemento–enamel junction (CEJ) with a 15 mm (height) segment of polyvinyl pipes (4mm) and then placed in deionized water. The dentin of these teeth was uncovered using a high-speed handpiece (KaVo Dental Corp., Biberach, Germany) with a 0.15-mm-thick diamond disc (D943-080, Kerr-Rotary, USA). These teeth were assigned randomly to three groups (CA, 5 wt.% β-TCP, and 10 wt.% β-TCP), with each group containing thirty-five teeth. Using distilled water (DW), the dentinal surface of these teeth was cleaned, followed by their etching (with 35% orthophosphoric acid) for 1 min and then washing with DW and drying. Using a micro-brush, the adhesives were applied to the dentin of these teeth. Air thinning for 3 s was carried out following the adhesive application, which was again followed by another use of the adhesive. Curing of the adhesive layer was then carried out using a dental curing light (Eliphar S10; 600 mW·cm−2 output; 3M ESPE, St. Paul, MN, USA), which was applied for 20 s from a distance of 10 mm. Post curing, a 2-mm layer of resin composite (Filtek Universal; 3M ESPE, St. Paul, MN, USA) was smeared onto each tooth, which was then photo-polymerized with the curing light. These bonded tooth specimens were stored at 37 °C for 1 day in deionized water. From each adhesive group containing thirty specimens, twenty specimens were analyzed for shear bond strength (SBS) analysis, five specimens were used to assess the bond reliability of the resin-dentin interface (by means of SEM) and five specimens were analyzed by FTIR and DC.

### 2.4. SBS Testing and Investigation of the Failure Modes

The teeth involved in SBS assessment were sectioned (similar to the steps of dentinal uncovering explained earlier) to form flat surfaces with an area of 5 mm × 5 mm. The specimens were polished for #240, #300 and #400 grit sandpaper for 30 s. SBS was assessed using Instron universal testing machine (Instron 5965 material testing system) using a knife-shaped probe at a crosshead speed of 5 mm/min with a standard load cell until specimen fracture. The SBS was presented in mega-pascals (MPa).

Pre-testing, ten tooth specimens from each adhesive group that was designated for SBS assessment were thermocycled (TC) inside a thermocycler (THE-1100, SD Mechatronik GmbH, Feldkirchen-Westerham, Germany). The remaining ten samples remained non-thermocycled (NTC). The TC samples received 10,000 cycles in water baths with a temperature range between 5–55 °C for 30 s, and a dwell time of 5 s was applied. All the TC and NTC specimens were then retained in deionized water for 21 days. The failure modes were investigated following the previous recommendations of Al-Hamdan et al. 2020 [22]. These failures were classified as adhesive, cohesive, or mixed type failures. Adhesive failures were defined as failure at the interface between the bonding agent (adhesive) and the adherend (dentin). A failure was considered cohesive when a layer or part of the adhesive remained on the dentin or resin surfaces. Mixed failures were identified when part of the substrate, i.e., dentin or resin, remained attached to the other half after intended bond failure.

### 2.5. Adhesive–Dentin Interface Analysis 

The samples for the analysis of the adhesive–dentin interface (*n* = 5) were prepared following the steps of our earlier study [23]. Teeth were sectioned in the bucco-lingual direction using a slow-speed lubricated diamond saw (KaVo Dental Corp., Biberach, Germany). Specimens were polished on 600 and 1200 grit abrasives, (Beuhler Polisher, Lake Bluff, IL, USA), followed by ultrasonic bath cleaning in distilled water (Bandelin Digital, Sigma-Aldrich Darmstadt, Germany). The specimens were then fixed on aluminum stubs, coated with a gold layer, and viewed in an SEM (JEOL JEM-1400 series 120kV-USA).

### 2.6. FTIR Spectroscopy and DC Investigation

FTIR spectroscopy was employed to evaluate DC of adhesives (CA, β-TCP 5 wt.% and β-TCP 10 wt.%). Spectra were obtained and analyzed before and after curing the group resins. Adhesives were applied on the potassium bromide disc of the spectroscope (Thermo Scientific Nicolet 6700 FTIR spectrometer, MA, USA). Peaks for C-C double bonds were recorded for the unpolymerized and photo polymerized resin (40 s). Aromatic (C-C) and aliphatic (C=C) reference peaks recorded at 1607 cm^−1^ and 1638 cm^−1^ were collected. The percentages of unreacted double bonds (absorbance intensities of C=C and C-C) were evaluated using the following formula.

DC = [1′ − (C aliphatic/C aromatic)/(U aliphatic/U aromatic)] × 100%(1)

where, C aliphatic is at the 1638 cm^−1^ peak of the cured resin, C aromatic is at the 1607 cm^−1^ peak of the cured resin, U aliphatic is at the 1638 cm^−1^ peak of the uncured resin, and U aromatic is at the 1607 cm^−1^ peak of the uncured resin.

### 2.7. Statistical Analysis

The results of the SBS testing and DC analysis were assessed using SPSS-20.0 (IBM, Chicago, IL, USA). ANOVA and post hoc multiple comparison tests were used for the analysis (significance level < 0.01).

## 3. Results

### 3.1. Outcomes of the Filler Nanoparticle’s Characterization

**Figure 1:**
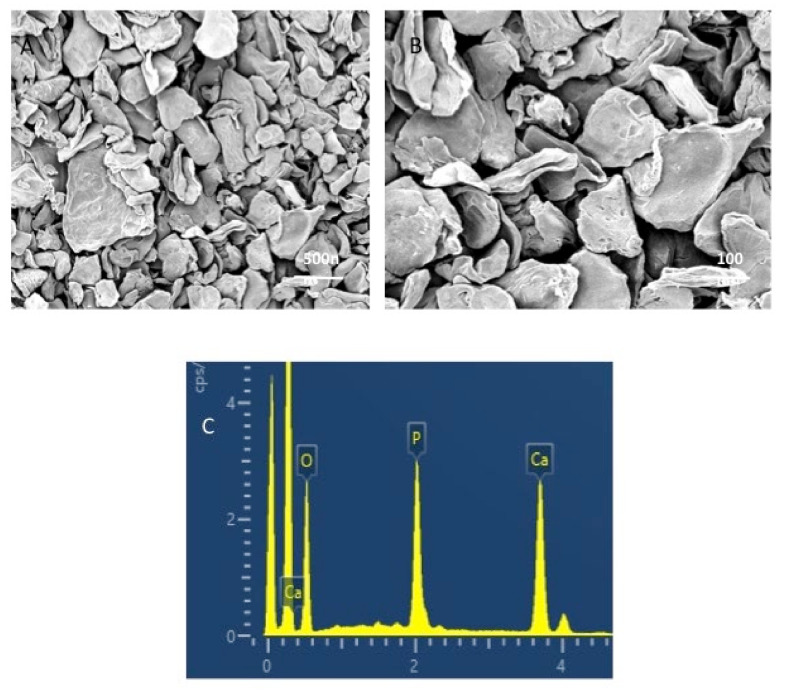
Representative (**A**) low- and (**B**) high-magnification SEM images of β-tricalcium phosphate particles (β-TCP). The particles indicated agglomerated morphology of irregular sized polygonal crystals ranging from 300 nm to 600 nm. (**C**) Representative electron spectrum particles showing no impurities and mainly containing oxygen ‘O’ (24.2%), phosphorus ‘P’ (17.4%) and calcium ‘Ca’ (60.1%).

**Figure 2:**
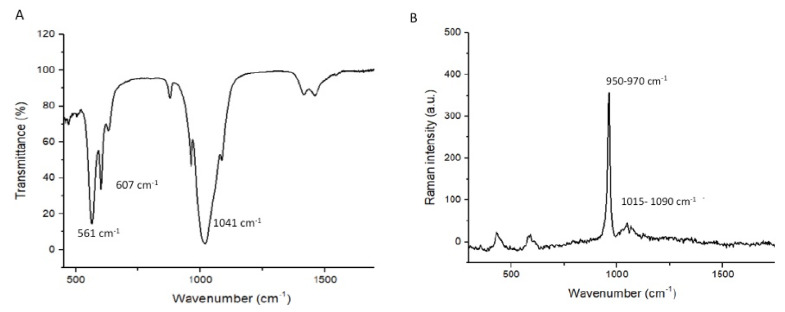
The FTIR analysis of the β-tricalcium phosphate is shown in (**A**). The band at 1041 cm^−1^ indicates the stretching mode of PO_4_ ^3−^ group and peaks at 561 and 607 cm^−1^ represent PO_4_^3−^ in β-TCP. (**B**) Raman spectrum of tricalcium phosphate indicates the internal vibration of the PO_4_^3−^ groups. The symmetric stretching (ν1) of P-O bonds show peaks at around 950 cm^−1^ and 970 cm^−1^. At 1015—1090 cm^−1^ range, asymmetric stretching (ν3) was observed.

The morphological characteristics of the β-TCP nanoparticles are shown in Figure 1A,B (low- and high-magnification SEM micrographs). The β-TCP nanoparticles demonstrated agglomeration with irregular shapes (Figure 1A,B). These agglomerated β-TCP nanoparticles were of various sizes, ranging between 500 and 1000 nm. The EDX analysis demonstrated the existence of oxygen (O) (24.2%), phosphorus (P) (17.4%), and calcium (Ca) (60.1%) (Figure 1C). The FTIR analysis of the filler nanoparticles demonstrated the stretching mode of PO4^3-^ which formed a strong band observed at 1041 cm^−1^. The vibration peaks of PO4^3−^ were observed at 561 cm^−1^, and 607 cm^−1^ (Figure 2A). The characteristic micro-Raman spectra of the β-TCP nanoparticles showed (Figure 2B) that symmetric (*v_1_*) and asymmetric stretching (*v_3_*) were detected. The *v_1_* vibration of the P-O bonds of tetrahedron is associated with peaks witnessed near 950–970 cm^−1^. The asymmetric stretching was at low intensity and observed at the 1015–1090 cm^−1^ peaks (Figure 2B).

### 3.2. Outcomes of the SBS and Failure Mode Analysis

The outcomes of the μTBS are shown in Table 1. The 10 wt.% β-TCP adhesive presented the highest μTBS values (NTC-10 wt.% β-TCP: 33.55 ± 3.73 MPa, TC-10 wt.% β-TCP: 30.50 ± 3.25 MPa) followed by the 5 wt.% β-TCP adhesive (NTC-5 wt.% β-TCP: 32.37 ± 3.10 MPa, TC-5 wt.% β-TCP: 27.75 ± 3.15 MPa), while the CA presented relatively low μTBS values (NTC-CA: 27.34 ± 3.11 MPa, TC-CA: 24.70 ± 3.64 MPa) (Table 1). All the comparisons within the same group demonstrated statistically significant differences (*p* < 0.01). Among the inter-group evaluations, statistically significant differences (*p* < 0.01) were only observed when the CA values for both NTC and TC samples were compared with both the nanoparticle reinforced adhesive groups (Table 1).

Adhesive type failure was most commonly found in our study (>80%) (Table 1). None of the adhesive groups presented any cohesive failure. The CA group did not demonstrate any mixed failure, while the nanoparticle-reinforced groups demonstrated mixed type failures as well (ranging between 10–20%).

### 3.3. Outcomes of the Analysis of the Adhesive–Dentin Interface

**Figure 3:**
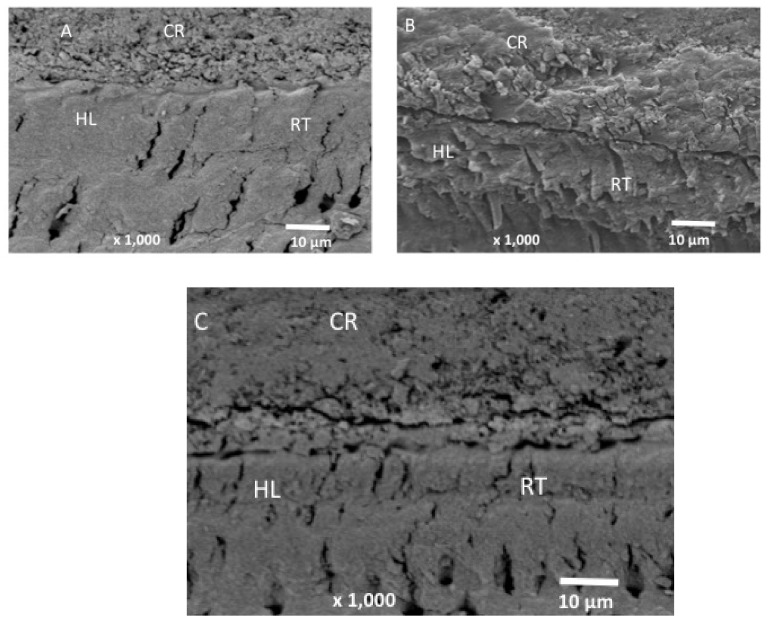
SEM images of the bonded resin and dentin interface using (**A**) control dentin adhesive, (**B**) 5 wt.% and (**C**) 10 wt.% tricalcium phosphate dental adhesive. All specimens formed a uniform adhesive layer that bonded well to the composite resin (CR). The formation of a hybrid layer (HL) was observed along with resin tags (RT). Gaps within the hybrid layer and the adhesive can also be observed in the unmodified dentin adhesive specimen. 5 wt.% adhesive exhibited longer and more numerous resin tags, with the formation of fewer gaps within the hybrid layer. (**B**) The 10 wt.% presented a uniform hybrid layer with gaps at the resin–dentin joint and shorter resin tags.

SEM micrographs showing the bonded resin dentin interface are presented in Figure 3A (CA), Figure 3B (5 wt.% β-TCP), and Figure 3C (10 wt.% β-TCP). It can be observed that all the specimens were able to form a thick uniform adhesive layer that bonded well to the composite resin. For the CA sample, comparatively shorter resin tags with few gaps were seen over the hybrid layer (Figure 3A). For the 5 wt.% β-TCP adhesive, resin tags that were greater in both length and number were seen, with fewer gaps within the hybrid layer (Figure 3B). For the 10 wt.% β-TCP adhesive, a uniform hybrid layer without any gaps at the resin dentin interface was seen with a smaller number of resin tags (Figure 3C).

### 3.4. Outcomes of the FTIR and DC Analysis

**Figure 4:**
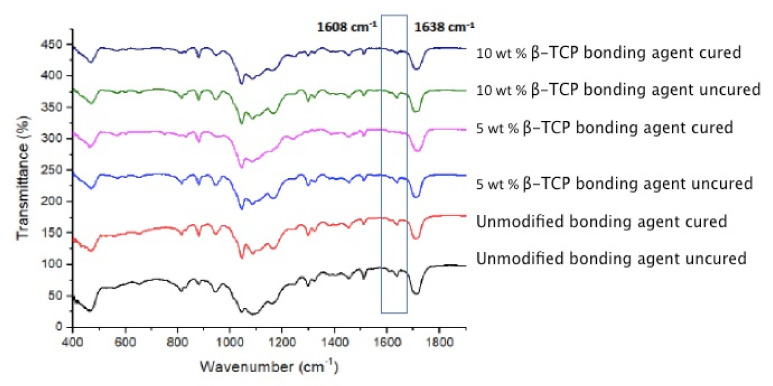
FTIR spectra of polymerized and unpolymerized groups from unmodified and BTCP nanocrystal-modified adhesive. The degree of conversion was calculated by estimating the changes in peak height ratio of the absorbance intensities of aliphatic C=C peak at 1638 cm^−1^ and that of an internal standard peak of aromatic C=C at 1608 cm^−1^ during polymerization, in relation to the uncured adhesive as indicated with the box.

**Figure 5:**
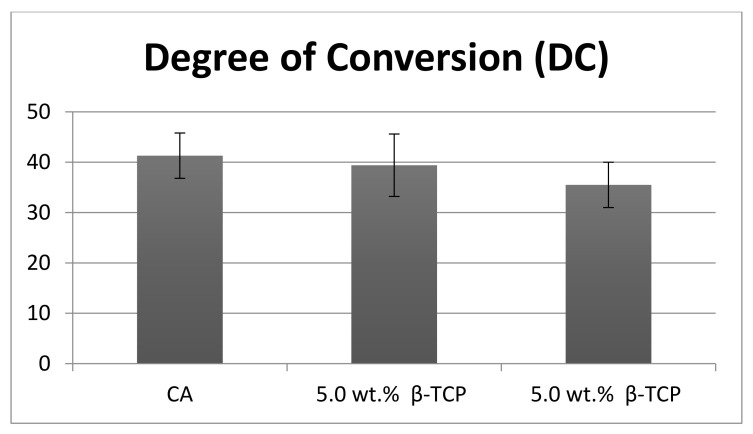
Comparison of DC among the study groups. CA: control adhesive.

The representative FTIR spectra of the polymerized and unpolymerized adhesives belonging to the three groups (CA, 5 wt.% β-TCP, and 10 wt.% β-TCP) were collected and then merged together in Figure 4. The estimation of the DC was performed by calculating the discrepancies in the peak height ratio of the absorbance strengths of aliphatic C=C peak at 1638 cm^−1^ and that of a standard inner peak of aromatic C=C at 1608 cm^−1^ during polymerization, as related with the unpolymerized adhesive, represented by a lined box in Figure 4. As can be seen from the DC results (Table 2 and Figure 5), the CA group demonstrated the highest DC (41.3 ± 4.5), followed by the 5 wt.% β-TCP group (39.4 ± 6.2), while the lowest DC was observed for the 10 wt.% β-TCP group (35.5 ± 3.9). No statistically significant findings (*p* > 0.01) were perceived when comparing the DC values of the CA and 5 wt.% β-TCP adhesive groups or when comparing those of the 5 wt.% β-TCP and 10 wt.% β-TCP adhesive groups. Significant differences (*p* < 0.01) were only observed when the DC values of the CA group were compared with those of the 10 wt.% β-TCP group (Table 2).

## 4. Discussion

On the basis of our study findings, the hypothesis that the integration of β-TCP nanoparticles in the adhesive composition would improve its adhesive bonding, dentin bond strength, and degree of conversion was partly accepted. We observed an improved SBS and dentin penetration, and a compromise in DC for the adhesives containing β-TCP nanoparticles. Several earlier studies have conveyed similar findings and reported that the incorporation of inorganic fillers into adhesives results in an improvement in its mechanical properties [23,24,25]. Two important ions that are part of the normal tooth structure and play a vital role in reinforcing its structural integrity are calcium and phosphate [26]. In the case of demineralization, the presence of these two ions ensures that the tooth structure can be remineralized promptly [27]. As composite restoration regularly suffers from micro-leakage, the occurrence of calcium and phosphate ions ensures efficient remineralization of the tooth, thus averting the effects of micro-leakage [28]. The β-TCP nanoparticles enjoy high biocompatibility and perform optimally when in a physiological environment like the oral cavity [20]. In line with these advantages, it was decided in our study to incorporate these filler nanoparticles in the adhesive and to appraise the effect of their integration on different properties of the adhesive.

On SEM, the filler nanoparticles demonstrated an irregular shape with agglomeration (Figure 1A,B). This morphology is typical for β-TCP nanoparticles, as demonstrated by previous studies [21,29]. The EDX analysis of these nanoparticles demonstrated the presence of oxygen, phosphorus, and calcium (Figure 1C). As explained earlier, calcium and phosphorus are part of the composition of the tooth [30], and they can remineralize the tooth instantaneously after acidic challenge [26]. The FTIR spectra (Figure 2A) displayed characteristic bands for β-TCP nanoparticles, and the micro-Raman spectra (Figure 2B) also displayed characteristic peaks related to these filler nanoparticles, similar to several previous studies [21,30,31].

The characterization of the adhesive–dentin interface yielded interesting results. It was seen that all the adhesives (CA, 5 wt.% β-TCP, and 10 wt.% β-TCP) formed a thick adhesive layer, which bonded well with the composite resin (Figure 3A–C). However, several gaps were seen in the hybrid layer observed for the CA, while the nanoparticle-reinforced adhesives demonstrated no to only a few gaps in the hybrid layer. The CA sample also demonstrated shorter resin tags compared to the other two adhesives (on visual observation). Though we did not evaluate the resin tags length inside the tubules in our study, it should be kept in mind that their dissemination depth does not significantly impact the adhesive’s bond strength [32].

In the present study, the bond strength of the adhesive was tested using SBS testing. This is a reliable technique for testing the material attachment strength with the dentin and has been used in several previous studies [33,34]. Previously, a former study recommended that although the addition of inorganic fillers in the adhesive is useful, their wt.% when being added should not be >10%, as that would result in a compromised bond strength [35]. In line with this recommendation, we did not surpass the 10 wt.% of filler nanoparticles in our adhesives. The inclusion of TCP-based nanoparticles in the adhesive triggered an increase in the bond strength for these reinforced adhesives [36]. Materials with remineralizing ions in their composition discharge them sporadically [37], and this could possibly have enhanced the μTBS of the β-TCP-containing adhesive in the present study. It has been shown before that remineralizing ions containing materials can biomineralize with the collagen fibers of the dentin [8], producing a stronger adhesive–dentin bond, and this could be attributed to the increased SBS of the β-TCP containing adhesive in our study. Concerning interfacial fractures, most of the failures observed in our study were of the adhesive type. This outcome is in agreement with a previous study, which also demonstrated adhesive type failure to be most common following integration of the filler nanoparticles in the adhesive [38]. Dentin is a collagenous wet tissue, and bonding with it is challenging [39], and this could also have played a part in the observance of this type of failure. The oral cavity is dynamic, and it regularly faces temperature fluctuations; therefore, to replicate this, we thermocycled specimens prior to SBS testing. ISO standard number 11,405 mentions that a temperature range of 5 to 55 °C is appropriate for inflicting aging on dental materials for a restricted time period [40]. Earlier studies have shown that TC causes a decline in the bond strength of the adhesive [41,42], and our μTBS findings are in harmony with these previous results.

The DC investigation revealed that the highest values were observed for CA, followed by 5 wt.% β-TCP and 10 wt.% β-TCP adhesives. Few previous studies have verified that the inclusion of filler nanoparticles in the adhesive, despite causing an appreciation in bond strength, also results in a lower DC in the nanoparticle-containing adhesives [23,43]. The results of the present study are in agreement with these previous findings, as we also observed a negative linear relationship between the DC and filler concentration. An increased DC is desirable in CA, as it guarantees that a sufficient quantity of monomers is polymerized [44], subsequently decreasing the micro-leakage and the possibility of the development of secondary caries. A plausible justification of this result could be that inorganic fillers are opaque and thus do not offer an adequate opportunity to the curing light to penetrate, thus producing a hindrance in the sufficient change of monomers into polymers, causing a lower DC [45,46].

One of the limitations of our study is that biocompatibility and antimicrobial activity of the nano particles used was not assessed. Biocompatibility and antimicrobial function are critical for dentin interaction and prevention of recurrent caries at the resin dentin interface. In addition, the oral cavity is dynamic and could present numerous challenges to foreign materials (adhesive or restoration). As the present study was in vitro in nature, the outcomes of the study should be regarded in light of the study setting. Therefore, further investigations assessing the cellular and bacterial interaction of β-TCP compositions in a dynamic environment are recommended

## 5. Conclusions

The incorporation of 5 and 10 wt.% concentrations of β-TCP particles resulted in an increase in SBS values. A linear decline in DC values was witnessed when the nanoparticle concentration increased. Further research focusing on exploring the influence of higher filler concentrations on adhesive properties are recommended.

## Figures and Tables

**Table 1:** Means and standard deviations for bond strength and failure modes among the study groups.

	SBS (MPa) (Mean ± SD)			Failure Mode Analysis (%)		
Group (*n* = 10)	NTC	TC	*p*-Value	Adhesive	Cohesive	Mixed
Unmodified CA	27.34 ± 3.11 ^a A^	-	<0.01	100	0	0
	-	24.70 ± 3.64 ^b A^		100	0	0
5.0 wt.% nano-β-TCP	32.37 ± 3.10 ^a B^	-		80	0	20
	-	27.75 ± 3.15 ^b B^		80	0	20
10.0 wt.% nano- β-TCP	33.55 ± 3.73 ^a B^	-		90	0	10
	-	30.50 ± 3.25 ^b B^		80	0	20

Dissimilar superscript small alphabets in the same row denote statistical difference. Dissimilar superscript capital alphabets in the same column denote statistically significant difference.

**Table 2:** Degree of conversion (DC) displayed by CA, β-TCP 5%, and β-TCP 10% adhesive.

Group	Degree of Conversion (Mean ± SD)	Tukey Test (*p* < 0.01) *
Unmodified CA	41.3 ± 4.5	A
5.0 wt.% β-TCP	39.4 ± 6.2	AC
10.0 wt.% β-TCP	35.5 ± 4.5	C

β-TCP: β-tricalcium phosphate-modified experimental adhesive; CA: experimental adhesive (control). * Dissimilar uppercase letters in this column indicate statistically significant difference among the groups (CA, 5 wt.% β-TCP, and 10 wt.% β-TCP).

## Data Availability

Data of the study is available on request form the corresponding author.
